# Phosphate decreases urine calcium and increases calcium balance: A meta-analysis of the osteoporosis acid-ash diet hypothesis

**DOI:** 10.1186/1475-2891-8-41

**Published:** 2009-09-15

**Authors:** Tanis R Fenton, Andrew W Lyon, Michael Eliasziw, Suzanne C Tough, David A Hanley

**Affiliations:** 1 Clinical Nutrition, Alberta Health Services, Calgary, AB, Canada; 2 Department of Community Health Sciences, University of Calgary, Calgary, AB, Canada; 3 Department of Pathology & Laboratory Medicine, University of Calgary, Calgary, AB, Canada; 4 Calgary Laboratory Services, Calgary AB, Canada; 5 Department of Medicine and Oncology, University of Calgary, Calgary, AB, Canada

## Abstract

**Background:**

The acid-ash hypothesis posits that increased excretion of "acidic" ions derived from the diet, such as phosphate, contributes to net acidic ion excretion, urine calcium excretion, demineralization of bone, and osteoporosis. The public is advised by various media to follow an alkaline diet to lower their acidic ion intakes. The objectives of this meta-analysis were to quantify the contribution of phosphate to bone loss in healthy adult subjects; specifically, a) to assess the effect of supplemental dietary phosphate on urine calcium, calcium balance, and markers of bone metabolism; and to assess whether these affects are altered by the b) level of calcium intake, c) the degree of protonation of the phosphate.

**Methods:**

Literature was identified through computerized searches regarding phosphate with surrogate and/or direct markers of bone health, and was assessed for methodological quality. Multiple linear regression analyses, weighted for sample size, were used to combine the study results. Tests of interaction included stratification by calcium intake and degree of protonation of the phosphate supplement.

**Results:**

Twelve studies including 30 intervention arms manipulated 269 subjects' phosphate intakes. Three studies reported net acid excretion. All of the meta-analyses demonstrated significant decreases in urine calcium excretion in response to phosphate supplements whether the calcium intake was high or low, regardless of the degree of protonation of the phosphate supplement. None of the meta-analyses revealed lower calcium balance in response to increased phosphate intakes, whether the calcium intake was high or low, or the composition of the phosphate supplement.

**Conclusion:**

All of the findings from this meta-analysis were contrary to the acid ash hypothesis. Higher phosphate intakes were associated with decreased urine calcium and increased calcium retention. This meta-analysis did not find evidence that phosphate intake contributes to demineralization of bone or to bone calcium excretion in the urine. Dietary advice that dairy products, meats, and grains are detrimental to bone health due to "acidic" phosphate content needs reassessment. There is no evidence that higher phosphate intakes are detrimental to bone health.

## Background

Phosphate is generously supplied in the diet through meat, grains and dairy products, and increasingly in recent decades, it is added to foods as food additives [1]. However, the understanding of dietary phosphate's role on bone health is not clear. While phosphate is a fundamental mineral component of hydroxyapatite, the principal structural element of bone, the acid-ash hypothesis posits that dietary phosphate, a marker of the metabolic production of acid, is detrimental to bone [2-4].

According to this acid-ash hypothesis, "acidic" ions such as phosphate contribute to the diet acid load (referred to as the renal acid load) which then the skeleton buffers, causing demineralization of bone, and bone calcium excretion in the urine, contributing to osteoporosis [2-4]. The acid-ash hypothesis proposes that the modern diet causes osteoporosis since the quantity of "acidic" ions (phosphate (PO_4_^---^), sulfate (SO_4_^--^), chloride (Cl^-^) in the diet is greater than the quantity of "alkaline" ions (sodium (Na^+^), potassium (K^+^), calcium (Ca^++^), and magnesium (Mg^++^)) [2-4]. Under the hypothesis, these ions are summed in the following equation to predict the potential renal acid load of the diet: (1.8PO_4_^--- ^+ SO_4_^-- ^+ Cl^-^) - (Na^+ ^+ K^+ ^+ 2Ca^++ ^+ 2 Mg^++^) [2].

Phosphate is considered to be the major dietary source of acid [2]. Many foods in the modern diet are considered detrimental to bone health, under the acid-ash hypothesis, due partially to their phosphate contents. These foods include meats, fish, dairy products [2,5-10], and grains [3,4,10], as well as many processed foods [3,4,10]. In contrast, this hypothesis posits that sodium is protective of bone health, which is not in agreement with concerns that sodium may compete with calcium for resorption in the kidney, and thus may compromise calcium metabolism and bone health [11,12]. The foods that are considered to protect skeletal mineral under this hypothesis are fruit and vegetables since these foods supply organic molecules that are metabolized to bicarbonate and therefore are considered "alkaline" [2-4,10,13,14].

Although the acid-ash hypothesis has been widely accepted and broadly stated as the major modifiable risk factor for bone loss in well cited scientific papers [4,15], as well as textbooks [16], reference works [17,18], and lay literature, this hypothesis has not been subjected to critical review. In spite of the lack of critical review, this hypothesis is heavily promoted to the public via the internet and other advertising for commercial gain, promoting products related to an alkaline diet.

The quantity of excess urinary calcium excretion associated with the acid-load of the modern diet is of sufficient quantity that could, if it is not accompanied by decreased fecal calcium losses, lead to the bone mineral loss of osteoporosis [19]. However, acid-generating diets are not detrimental to whole body calcium balance [20]. This recent finding [20] raises doubt about the acid-ash hypothesis which raises the additional question of whether an acid load from phosphate contributes to the excretion of bone calcium and the development of osteoporosis.

Additional variables may influence the relationship between dietary phosphate intakes and bone health. Calcium intakes, that is whether calcium intakes are limited or insufficient, might influence the relationship between phosphate, the diet acid load, and bone health [21,22]. As well, the general concept of the acid-ash hypothesis is based on the "acidity" of the diet, therefore the degree of protonation of the ions in the foods (e.g. H_3_PO_4 _versus Na_2_HPO_4_) may be of importance. A meta-analysis provides an opportunity to assess these issues.

The purpose of this study is to use the techniques of meta-analysis to quantify the potential contribution of dietary phosphate to bone health, as measured by surrogate and, if possible, direct measures of this osteoporosis. Second, it will assess whether these effects are altered by the level of calcium intake of the subjects and by the degree of protonation of the phosphate supplements.

## Methods

### Literature search for the systematic review

Literature relating to intervention studies of dietary phosphate supplementation on calcium metabolism was identified through computerized searches. We used four comprehensive search themes based on keywords/textwords, which were combined using the Boolean operators "OR" within the themes and then "OR" and "AND" to combine the themes. The themes were related to phosphate (phosphorus, phosphate, phosphates), acid excretion (net acid excretion, acid excretion, acid-base equilibrium), and the outcomes: bone health (bone, bones, bone density, bone mineral density, fractures, biopsy, bone resorption markers), or calcium metabolism and excretion (calcium, calcium, calciuria, excretion, urine, urinary, balance, retention). The search was limited to adults 19+ years, with no upper age limit. Databases searched included Medline back to 1966 (OVID and PubMed), Cochrane Database of Systematic Reviews, CINAHL back to 1982, EMBASE back to 1980, the Cochrane Controlled Trials Register, and , all up to January 2009. We developed our search strategy in PubMed and modified it for use in other databases. The literature search was not limited to English language articles. Reference lists were reviewed for additional relevant studies.

### Selection criteria for the literature

Intervention studies were included if they manipulated human subjects' dietary phosphate intake through supplemental phosphate salts and reported outcomes related to bone health, urinary calcium excretion, and/or calcium balance in healthy adult subjects. Outcomes related to bone health included: bone biopsy, change of bone mineral density (BMD) or fractures, bone resorption markers. Since the aim of this meta-analysis was to study the potential for the acid-ash diet hypothesis to have a role in the development of osteoporosis in apparently healthy adults, studies were restricted to adult subjects. We excluded studies of subjects with chronic conditions such as renal disease or conditions which could alter calcium absorption or excretion such as inflammatory bowel disease, cancer, weight loss, or decreased ambulation. Studies with an observational design were not included since they were likely to have confounded effects due to changes in protein, energy or calcium intakes, since these nutrients are correlated with phosphate in foods. Calcium balance was defined as calcium intake minus excretion (urinary plus fecal) [23].

### Methodological quality

We evaluated the studies' methodological quality in terms of whether the guidelines for calcium balance studies by the Institute of Medicine [24] were followed and whether the subjects were randomized to the interventions [25,26]. The Institute of Medicine recommends calcium balance studies control the subjects' calcium intakes for the seven or more days prior to measurement of the outcomes, provide of all the food to subjects, accurately measure the amounts of food consumed, and perform laboratory analysis to determine the calcium composition of the foods [24].

### Statistical Analyses

Multiple linear regression analyses were conducted to examine the effect of increased daily dietary phosphate on urinary calcium excretion, calcium balance, and markers of bone resorption or turnover (i.e., the percent changes of hydroxyproline and N-terminal telopeptide of Type I collagen). Interaction terms were included in the regression models to test the hypothesis that the estimated effects of the phosphate supplements were the same whether the subjects were on high or low calcium intakes or whether the phosphate supplement was acidic or not. The following variables were included in all the models to adjust for differences among the studies: age, gender, whether the studies were randomized, baseline phosphate intakes, and duration of the balance studies (Table 1 and Table 2). One study did not report the age of their subjects, who they described as "young women" and the median age of 22 was assumed to keep this study in the model. The analyses were weighted for study sample size.

**Table 1:** Included studies in the meta-analysis of calcium balance from a change of phosphate intake

**Study**	**Year**	**Subjects**	**Age range (years)**	**% female**	**Study design**	**Baseline** **PO4 intake (mmol/day)**	**Ethics approval**	**Food weighted **	**Food lab analysis**	**Usual Calcium intake**
Patton [21]	1953	young women	N/A	(100%)	RCO	25	N/A	yes	yes	no
Malm [27]	1953	male prisoners	20-56	0	CO	unclear	N/A	yes	yes	yes
Goldsmith [28]	1976	postmenopausal women with osteoporosis	63-75	7(100%)	CO	40	N/A	no	no	yes
Bell [29]	1977	young adults	24-36	3/8 (38%)	CO	32	yes	yes	yes	no
Spencer [30]	1978	adult men	38-65	0	CO	25	N/A	yes	yes	no
Hegsted [31]	1981	adult males	19-25	0	CO	33	yes	yes	yes	no
Zemel [32]	1981	young men	18-24	0	CO	27	yes	yes	yes	no
Schuette [33]	1982	young men	19-26	0	CO	29	yes	yes	yes	no
Spencer [34]	1986	adult males	48-71	0	CO	26	yes	yes	yes	no
Whybro [36]	1998	healthy men	19-38	0	RCO	26 & 32	yes	no	no	no
Kemi [37]	2006	young women	20-28	48(100%)	RCO	16	yes	no	no	no
Krapf [35]	1995	young men	22	0	CO	49	yes	no	no	no

**Table 2:** Study arm calcium intakes, phosphate doses, urine calcium and calcium balance

**Study**	**n**	**Phosphate dose (mmol/day)**	**Phosphate source**	**Calcium Intake (mmol/day)**	Days on Calcium intake*	Days on each balance study after adaption**	**Change uCalcium (mmol/day)**	**Change Calcium balance (mmol/day)**
Patton [21]	18	10	Na_2_HPO_4 _& Na glycerophosphate	9	7	7	-0.4	-0.2

Patton [21]	18	19	"	9	7	7	-1.0	0.6

Patton [21]	18	10	"	24	7	7	-0.4	-0.6

Patton [21]	18	19	"	24	7	7	-1.5	0.3

Patton [21]	18	10	"	39	7	7	-1.0	0.03

Patton [21]	18	19	"	39	7	7	-0.4	1.1

Malm [27]	4	24	H_3_PO_4_	-	98	7	-0.9	n/a

Malm [27]	2	32	H_3_PO_4_	11	98	28	-0.7	0.7

Malm [27]	4	26	H_3_PO_4_	20	98	28	-0.9	0.1

Malm [27]	2	19	H_3_PO_4_	13	56	56	-1	0.03

Goldsmith [28]	7(4)	32	K_2_H & KH_2_	21	7	4	-0.9	0.45

Bell [29]	5	37	Na PolyP	18	6	22	-1.7	n/a

Spencer [30]	10	37	Naglycerophosphate	5	0	22	-0.8	0.03

Spencer [30]	8	37	Naglycerophosphate	21	0	40	-1.7	0.70

Spencer [30]	3	39	Naglycerophosphate	36	0	34	-2.3	-0.35

Spencer [30]	6	36	Naglycerophosphate	50	0	31	-2.1	0.10

Hegsted [31]	8	49	KH+PO+	13	0	12	-2.5	0.94

Zemel [32]	8	32	KH_2_PO_4_	10	2	11	-2.0	2.7

Zemel [32]	8	32	(NaPO_3_)_6_	10	2	11	-2.0	1.3

Schuette [33]	8	25	KH_2_PO_4_	15	2	6	-0.9	0.03

Spencer [34]	1	35	Naglycerophosphate	6	0	66	-3.2	0.5

Spencer [34]	4	34	Naglycerophosphate	20	0	42	-3.0	1.3

Spencer [34]	2	41	Naglycerophosphate	34	0	33	-3.6	0.00

Spencer [34]	3	40	Naglycerophosphate	51	0	40	-3.5	0.2

Whybro [36]	9	32	NaH_2_PO_4_	25	5	2	-1.1	n/a

Whybro [36]	11	48	Not stated	25	5	9	-2.4	n/a

Kemi [37]	14	8	Na_2 _Na_3 _HPO_4_	6	0	1	-0.2	n/a

Kemi [37]	14	24	Na_2 _Na_3 _HPO_4_	6	0	1	-0.5	n/a

Kemi [37]	14	48	Na_2 _Na_3 _HPO_4_	6	0	1	-0.5	n/a

Krapf [35]	6	9.6	IV PO4 vs Cl	35	4	3	-3.5	n/a

* days on calcium intake prior to the measurement of outcomes

** the shortest number of days on the balance study is reported if it varied within the comparison interventions

The cut-point used to stratify the analysis by calcium intake was an intake greater than or equal to versus less than that intake considered adequate by the Institute of Medicine of 1000 mg/day (25 mmol/day) for adults aged 19 through 50 years; 1200 mg/day (30 mmol/day) for those aged 51 or older [24]. Phosphate supplements were stratified into acidic (e.g. H_3_PO_4_) or neutral/alkaline (e.g. Na_2_HPO_4_) categories. Stata, Version 10.1 (StataCorp, College Station, Texas, USA), was used for the data analyses.

## Results

### Description of studies

The literature search identified 32 dietary phosphate intervention studies. Twelve of the studies met all the inclusion criteria [21,27-37] (Table 1). Studies not included used study periods of less than 24 hours [38-42]; had more than one intervention performed [43-51]; reported no numerical results in the paper or in response to a written request [52-54]; had an observational design [55-57]; or the subjects had chronic conditions [22,58,59]. No non-English language papers met the criteria for acceptance.

All of the included studies except one arm within one study [27] noted that calcium intakes were controlled. One study used postmenopausal women with osteoporosis as the subjects, and they described these patients as having "roentgenographic and clinical diagnoses" of osteoporosis [28]. One study [31] had other outcomes reported in a second paper [6]. Phosphate was supplemented in all of the studies by providing a daily dietary phosphate salt (Table 2), and some provided some additional sodium with the phosphate. Two studies [32,35] controlled for changes in sodium intake from the phosphate supplement by reducing NaCl when the sodium and phosphate were provided. The subjects consumed their usual calcium intakes in two studies [27,28] while the other subjects consumed a specified calcium intake for zero to 14 days prior to collecting outcome data. Eight of the 12 studies weighted the food intake of the subjects and analyzed identical portions for calcium content (Table 1).

### Methodological quality

Only one of the studies used superior methodology: using both randomization [25,26] and the Institute of Medicine's recommendations for calcium balance studies [21] (Tables 1 &2).

### Study Outcomes

None of the phosphate supplementation studies assessed bone related outcomes such as changes in BMD, as measured using absorptiometry, or the incidence of fractures. One study [28] included changes in BMD as measured using bone biopsies of the subjects while on supplemental phosphate, but did not report biopsy results during the control phase.

Eleven studies assessed the effect of supplemental dietary phosphate salts administered orally, while one administered the phosphate intravenously [35], on urine calcium over 24-hours in healthy adults [21,27-34,36,37]. Eight of the studies examined calcium balance over one to 66 days [21,27,28,30-34] and one assessed calcium balance among only four of the seven subjects [28]. Six studies examined the effect of dietary phosphate supplements on bone turnover markers [29,31-33,36,37].

As the present study was designed to examine conditions similar to the modern diet, we did not include two intervention arms (one arm in each of two studies [30,34]), as the subjects were given very high calcium intakes (2700 & 2600 mg/day, 68 & 65 mmol/day). These are greater than the Tolerable Upper Limit of the Institute of Medicine Dietary Reference Intake for calcium [24], and therefore we considered them experimental. The remaining 30 intervention arms had outcomes related to calcium excretion, calcium balance and/or markers of bone metabolism among 269 subjects (Table 1 and 2).

### Results from the individual studies

All 30 interventions that increased dietary phosphate intake showed a decrease in urine calcium; 16 of these changes were statistically significant [28-37], six were not significant [27,34], six interventions in one study did not report statistical significance nor information that permitted statistical testing [21]. Of the 22 interventions of calcium balance, only one study [32] demonstrated a significant change in calcium balance from the phosphate supplement. The direction of this effect was a statistically significant increase in calcium retention in response to the dietary phosphate supplement [32]. Only three of the studies reported the change of net acid excretion in response to the phosphate supplements [6,31,33,35]. All three studies noted increases in net acid excretion (45, 21, and 41 mEq/day) in response to the phosphate supplements of 49, 25, and 10 mmol/day, respectively.

The hydroxyproline results in the individual studies in response to phosphate supplementation were as follows: Bell reported a significant increase in hydroxyproline of 20% (p < 0.0001) [29], while three studies reported non-significant decreases between 5 and 20% [31-33]. For the N-telopeptide results, in response to the phosphate supplements, both Whybro et al and Kemi et al saw non-significant changes from baseline (-1.5%, -5.3%, and 29%) [36,37].

### Regression analysis

The multiple regression analyses results are summarized in Table 3 and illustrated in the Figures.

**Table 3:** Regression analysis results

	**Urine calcium**				**Calcium balance**			
**Analysis**	***B*_1_**	**p-value for slope**	**p-value for interaction**	**R^2^**	***B*_1_**	**p-value for slope**	**p-value for interaction**	**R^2^**

Low calcium intakes (*B*_1_)	-0.012	0.001	0.19	0.612	0.048	< 0.001	0.82	0.390
					
High calcium intakes	-0.012				0.048			

Neutral or alkaline phosphate supplement (*B*_1_)	-0.008	0.037	< 0.001	0.645	0.086	< 0.001	< 0.001	0.457
Acidic phosphate supplement	-0.070	< 0.001			0.008	0.78		

*B*_1 _= slope coefficient from regression model

R^2 ^= the proportion of variance explained by the regression analysis

**Figure 1:**
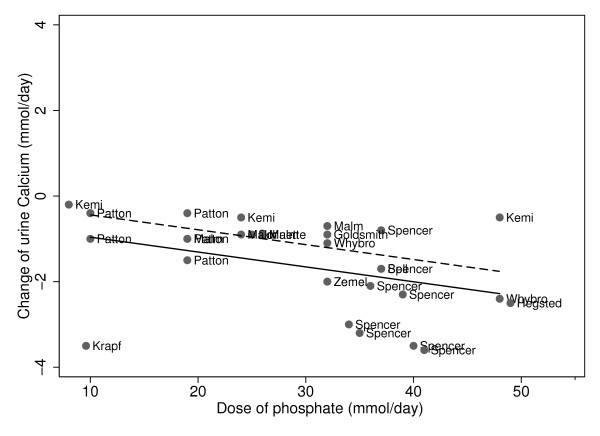
**Phosphate and change in urine calcium stratified by calcium intakes: Slope = -0.021 p = 0.001. Low calcium intakes: ------; High calcium intakes: ______**.

Urine calcium decreased as phosphate doses increased, regardless of calcium intakes. For both low and high calcium intakes, higher phosphate supplements led to significantly lower urine calcium excretion (Figure 1, Table 3, p = 0.001). The test of interaction, which assessed whether the effect of the phosphate supplement on urine calcium differed by calcium intakes of the subjects, revealed that the change (slope) in urine calcium was not significantly different when the calcium intakes were greater or less than the recommended intakes (p = 0.19). There was a slight difference in the quantity of urine calcium, between those on low versus high calcium intakes (Figure 1).

**Figure 2:**
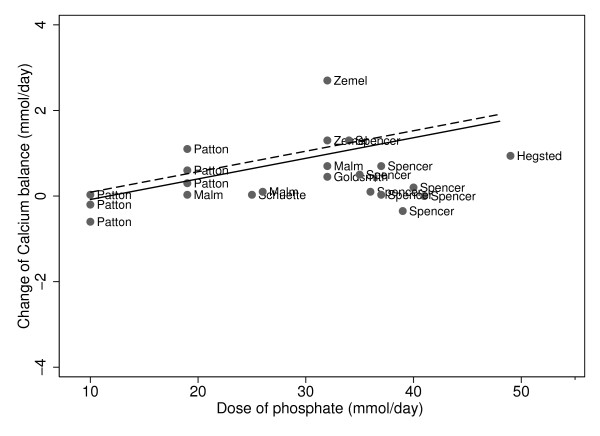
**Phosphate and change in calcium balance, stratified by calcium intakes: Slope = -0.048 p < 001. Low calcium intakes: ------; High calcium intakes: ______**.

In contrast, for both low and high calcium intakes, higher phosphate supplements led to significantly greater calcium balance (Figure 2, Table 3, p < 0.001). The test of interaction, which assessed whether the effect of the phosphate supplement differed by calcium intakes of the subjects, revealed that the change (slope) in calcium balance was not significantly different when the calcium intakes were greater or less than the recommended intakes (p = 0.82). There was no apparent difference in the quantity of calcium balance between those on low versus high calcium intakes (Figure 2).

**Figure 3:**
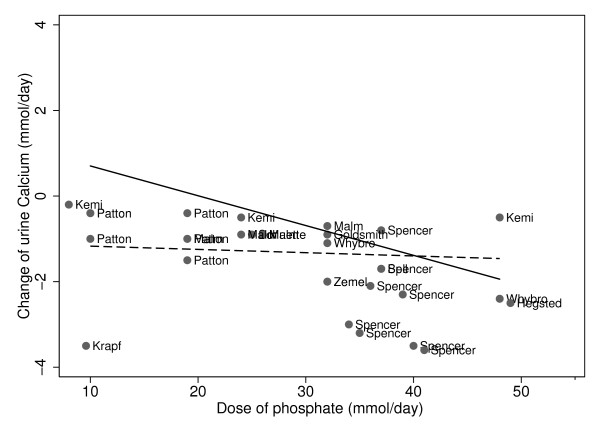
**Phosphate and change in urine calcium, stratified by composition of phosphate supplements: Slope for acidic = -0.070 p < 0.001; slope for neutral/alkaline = -0.008 p = 0.037. Acidic phosphate supplement: ______; Neutral/alkaline phosphate supplement:------**.

Urine calcium decreased as phosphate doses increased, whether the phosphate supplement was neutral/alkaline versus acidic (Figure 3, Table 3). When the phosphate supplement was neutral/alkaline, urine calcium decreased by 0.008 mmol/day for every mmol/day increase of phosphate dose (p = 0.037). The magnitude of change of urine calcium in response to the phosphate was significantly greater if the phosphate was an acidic salt (p < 0.001). Urine calcium decreased by 0.07 mmol/day for every mmol/day of phosphate supplement, when the phosphate was acidic (p < 0.001).

**Figure 4:**
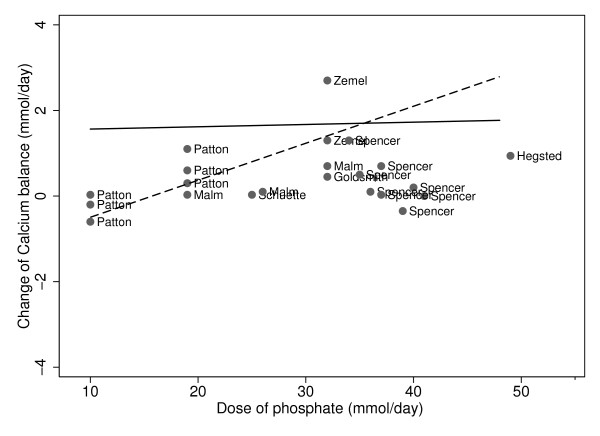
**Phosphate and change in calcium balance, stratified by composition of phosphate supplements: Slope for acidic = 0.008 p-value = 0.78; slope for alkaline/neutral = 0.086 p-value < 0.001. Acidic phosphate supplement: ______; Neutral/alkaline phosphate supplement:------**.

The effect of the degree of protonation of the phosphate supplements altered their effect on calcium balance (Figure 4, Table 3). When the phosphate supplement was neutral/alkaline, calcium balance increased by 0.086 mmol/day for every mmol of phosphate supplement (p < 0.001). The magnitude of change of calcium balance was significantly less when the phosphate was provided as an acidic salt (p < 0.001). Calcium balance increased non-significantly, by 0.008 mmol/day for every mmol/day of phosphate supplement, when the phosphate was acidic (p = 0.78).

**Figure 5:**
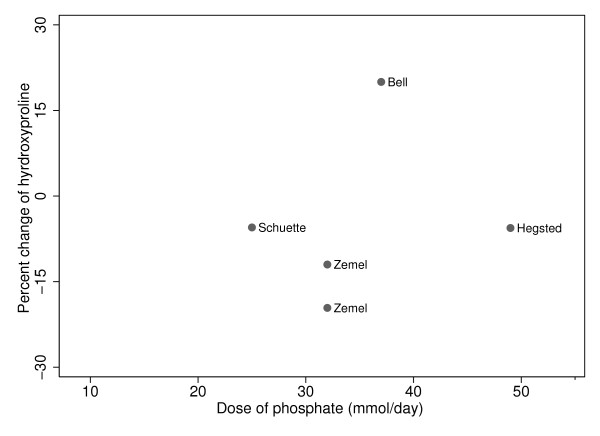
**Phosphate and change in markers of bone metabolism: Hydroxyproline**.

**Figure 6:**
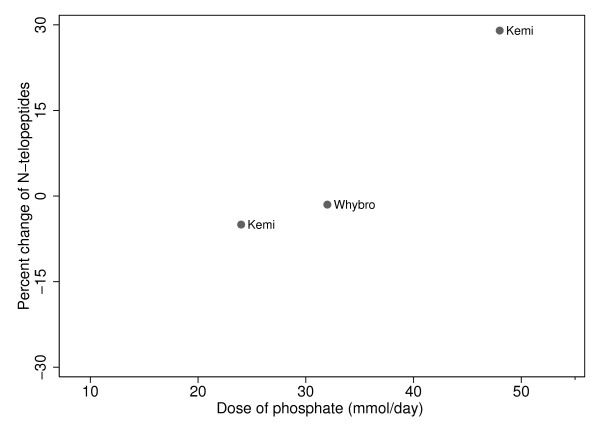
**Phosphate and change in markers of bone metabolism: N-telopeptide**.

As there were very few studies and the change of phosphate intake on the change of bone turnover markers, hydroxyproline and N-terminal telopeptide, did not appear to have straight line relationships, no attempt was made to combine these data in regression analyses (Figures 5 and 6).

## Discussion

All four of the urine calcium meta-analyses demonstrated that when dietary phosphate was increased, urine calcium decreased, whether the subjects had low or high calcium intakes and whether the phosphate supplement was neutral/alkaline or acidic. Three of the four calcium balance analyses revealed that as phosphate supplements are increased, calcium balance increased. However, when the phosphate salt was acidic, there was no important change in calcium balance. The effect of phosphate supplementation on the bone metabolism markers did not provide any clear information. The increased net acid excretion from phosphate supplements in three studies was associated with lowered urinary calcium excretion.

All of the findings of this study contradict the acid ash hypothesis since the hypothesis posits that an "acidic" ion such as phosphate causes increased urinary excretion of bone calcium, and therefore calcium balance would be decreased by phosphate. None of the study findings support the hypothesis since phosphate supplements did not increase urine calcium or decrease calcium balance. Dietary phosphate is considered to be the major source of excreted acid, followed by sulfate derived from protein [2]. However, this current study contradicts the assertion that dietary phosphate is detrimental to bone, and our previous work contradicts the assertion that protein is detrimental to bone [20]. When the evidence regarding the acid-ash hypothesis is critically examined, there is more contradiction than support for the hypothesis.

Most of the evidence in favor of the acid-ash hypothesis is based on the outcomes of changes of urinary calcium and changes of indicators of mineral metabolism or bone turnover markers [2,6,8,9,15,31,60-64]. However, urinary calcium and bone/mineral markers, are not direct measures of osteoporosis. Changes in urine calcium excretion are poor surrogate measures of bone health, as intestinal calcium absorption [65] and/or secretion [66] may also be altered by an intervention. Calcium balance changes are much better measures of changes of calcium status than changes of urinary calcium, and calcium balance studies can provide useful information about whole body calcium metabolism when the studies are well designed [24].

What better bone health outcomes have studies used as to test the association of the acid-ash hypothesis? The National Institutes of Health Consensus Panel defined Osteoporosis as ". a skeletal disorder characterized by compromised bone strength predisposing a person to an increased risk of fracture" [67]. The only currently available measures that address bone strength are the occurrence of low trauma fractures and/or biomechanical testing of bone biopsy material [68,69]. In the assessment of pharmaceutical interventions for osteoporosis, fracture prevention is considered the appropriate outcome measure for clinical trials, and not change in bone density. In clinical practice, however, bone density is an objective measure often used for following an individual's response to an intervention. Although low bone density is used in the clinical diagnosis of osteoporosis, it is simply a risk factor for bone fragility.

Two randomized controlled studies of the acid ash hypothesis have used changes of BMD as the outcome measure [70,71]. Both of these studies tested the BMD response to potassium citrate [70,71] and one of these studies also increased fruit and vegetable intakes in an additional study arm [71]. The results of these studies were opposite, one observed a decreased loss of spine BMD among the potassium citrate arm [70], while in the other study BMD change did not differ significantly between study arms [71]. This discrepancy may be explained by the use of allocation concealment, an indicator of study rigor [72]. The latter study that saw no differences in BMD used adequate allocation concealment [71] while the former study that saw a difference did not conceal their allocation [70]. Inadequate allocation concealment is associated with an overestimation of effect [72], therefore it is possible that the results from the former trial [70] overestimated the effect of potassium citrate on bone.

Three prospective observational cohort studies found some associations that appear to support the acid-ash hypothesis, using changes in bone mineral density as the outcome [73-75]. However, the three prospective studies with positive associations had inconsistent findings, since for each group in which a positive finding was identified there were other subject groups and/or bone sites for which the finding did not apply. The Framingham Osteoporosis study reported that in men potassium intake was associated with loss of bone mineral density at the femoral neck and trochanter, but not at the radius, and there were no associations found for women [74]. Fruit and vegetable intakes were not significantly associated with the changes of bone mineral density at any site for men or women in this study [74]. Of 12 associations assessed in this study regarding the acid-ash hypothesis, two were significantly associated while 10 were not [74].

In the EPIC-Norfolk study, vitamin C was associated with less loss of bone mineral density at the total hip among elderly women but not among elderly men, and only after the vitamin C intakes were divided into tertiles [75]. In the Aberdeen Prospective Osteoporosis Screening study, intakes of calcium and phosphorus (positively) and fat (negatively) were associated more strongly with the loss of bone mineral density over time than was potassium [73].

The two recent prospective cohort studies of the acid-ash hypothesis used fractures as the outcome measure. Fractures are considered a direct and clinically valid measure of osteoporosis [68,69]. A study among 1865 vegetarian and omnivorous peri- and postmenopausal women found that the risk of wrist fracture decreased significantly as protein intakes increased, whether the protein was from plants or meat [76]. This study provides further support for the concept that dietary protein is supportive of bone health [77,78]. A French study among 36,217 women examined the association between protein or the diet acid load on fractures (excluded high trauma or metastases fractures) found no overall support for the acid ash hypothesis as neither protein nor the diet acid load were associated with fracture risk [79]. However, in a subgroup analysis, both higher protein intake and diet acid load were associated with higher fracture risk among women in the lowest quartile of calcium intake [79]. In comparison, in this meta-analysis we found no important differences between phosphate intakes and calcium metabolism between those with low or high calcium intakes. In summary, the findings from prospective cohort studies of the acid-ash hypothesis of bone health demonstrated few findings consistent with the hypothesis, and numerous findings that did not support the hypothesis. It must be remembered that observational studies do not have the rigor of randomized control trials, and the findings from observational studies can be confounded or confused by other related variables. An example that might confound observational studies of nutrition and bone health could be due to those subjects who eat more fruit and vegetables may also get more bone protective exercise.

The acid-ash hypothesis posits that meats, grains and dairy products are detrimental to bone health due to their phosphate contents [16,3,4,10], however, this meta-analysis revealed that higher dietary intakes of phosphate do not increase either urinary calcium excretion or whole body calcium loss. Further to question the acid ash hypothesis, the evidence regarding higher net acid excretion from changes in dietary protein type or amount does not support the hypothesis [20]. No studies have evaluated the effect of grain foods on bone health. Dairy products, an important source of dietary phosphate, are also an important dietary calcium source [80] and an inexpensive source of high quality protein. Protein has been found to be supportive of bone health in a prospective cohort study and a randomized controlled trial [77,78]. Considering that this study has not upheld the concept that higher intakes of phosphate are detrimental to bone mineral maintenance, the consideration of meats, grains and dairy products as detrimental to bone health on the basis of their phosphate content must be questioned. Additionally, these foods that produce acid on metabolism are important sources of nutrients that are important for bone health, including calcium [81] (dairy products), protein [82,83] (dairy products and meats), and vitamin D [84] (some dairy products).

The recommendation for calcium balance studies by the Institute of Medicine to have study subjects consume the study calcium intake for at least a week prior to the outcome measurement [24] is designed to allow the subjects to adapt to the experimental calcium intake [24]. This adaption to the study calcium intake is important to lessen the chance that the adaption confuses or biases the effect of the intervention. Randomization of the interventions is very important in calcium balance cross-over studies since the adaptations to the calcium intake would be uneven in the two arms of before-after non-randomized cross-over studies.

Overall, the methodological quality of the studies of phosphate supplementation was poor since only one of the studies used superior methodology [21]: having randomization and using all of the Institute of Medicine's recommendations for calcium balance studies. Therefore, there is a chance that the results of the other studies are biased due to poor methodology. The magnitude of this bias would vary depending on whether the study calcium intakes were greater or less than the subjects' usual intakes. In spite of the questionable methodological quality, all of the other meta-analyses were in congruence with the one study [21] that used superior methodology, which suggests that the bias may not have been severe.

Concern has been raised about the phosphate, as well as caffeine, contents of cola soft drinks and their potential deleterious effects on bone health. Observational studies that measured BMD [85] or asked subjects about previous fractures [86] in relation to carbonated beverage intake documented associations between these beverages and poorer bone health. As well, a non-randomized cross-over intervention study identified higher bone resorption markers from colas compared to milk [87]. The results from this meta-analysis does not support the concept that the phosphate in soda is deleterious, therefore, these potentially deleterious effects could be due to lower milk consumption, and therefore protein, calcium or other nutrient intakes [87] since those that consume more carbonated beverage drink less milk [88,89], however generalizations from the studies on soft drinks are limited by the study designs used.

This meta-analysis has three strengths: First, this is the first study to systematically assess the calcium balance literature in response to changes of phosphate supplementation. Second, this meta-analysis includes an evaluation of methodological quality, and therefore an assessment of potential confounding or bias. Third, the stratified analyses allowed assessment of the effect of calcium intakes and the degree of protonation of the phosphate supplement on the findings.

This meta-analysis also has limitations. First, although calcium balance is neither a direct measure of bone health nor of the progression of osteoporosis, but rather it is a surrogate measure of this disease progression. Therefore this study lacks a direct measures of bone health. However, calcium balance is important since prolonged negative calcium balance leads to bone loss. Second, this study is limited by the poor methodological quality of the majority of the studies of phosphate intake on calcium metabolism.

In conclusion, this meta-analysis revealed that raising phosphate intakes decreases urine calcium and slightly increases calcium retention over a broad range of calcium intakes. This work does not support the acid-ash hypothesis concepts that "acidic" ions such as phosphate contribute to demineralization of bone and bone calcium excretion in the urine. A definitive study is needed that follows all of the recommendations for methodological quality for both calcium balance studies [24] as well as recommendations for intervention studies [25,26], with measurement of outcomes that are direct measures of bone strength, to determine whether or not there is an association between phosphate intake and osteoporosis.

## Competing interests

The authors declare that they have no competing interests.

## Authors' contributions

The author's responsibilities were as follows: TRF & AWL designed the study, TRF searched the literature, extracted the data, performed the statistical analysis and graphic representation and wrote the manuscript, ME directed the study's statistical analysis and graphic representation, AWL contributed to data analysis and writing of manuscript, SCT & DAH helped design the study and interpret the findings. All authors read and approved the final manuscript.
